# The complete mitochondrial genome of *Pleurocorallium inutile* (Octocorallia: Scleralcyonacea: Coralliidae)

**DOI:** 10.1007/s11033-025-10583-3

**Published:** 2025-06-01

**Authors:** Kenji Takata, Masanori Nonaka, Fei Xia, Taisei Kikuchi, Kodai Gibu, Nina Yasuda

**Affiliations:** 1 Graduate School of Agricultural and Life Sciences, The University of Tokyo, Yayoi, Tokyo, Japan; 2 Okinawa Churashima Foundation Research Institute, Motobu, Okinawa Japan; 3 Graduate School of Frontier Sciences, The University of Tokyo, Kashiwa, Chiba Japan

**Keywords:** Deep-sea coral, Precious coral, Genome skimming, Mitogenome assembly

## Abstract

**Background:**

Although the genus *Pleurocorallium* contains an economically valuable species, limited genetic information is available. Here, we report the complete mitochondrial genome sequence of *Pleurocorallium inutile*. In Japan, this species is known as damesango, meaning “useless coral”, because it has no commercial value when caught as a bycatch during precious coral harvesting. Although several species of *Pleurocorallium* can be distinguished morphologically, some cannot be differentiated using the limited number of characterized genetic regions, such as eight mitochondrial and elongation factor genes. *P. inutile* is one such species, and therefore, analyzing its complete mitochondrial genome will be useful for identifying its phylogenetic status, distinguishing it from other commercially important species, and providing insights into the evolution of octocoral mitochondrial genes.

**Methods and results:**

The 18,822 bp mitochondrial genome of *P. inutile* consists of 14 protein-coding genes, two ribosomal RNA (rRNA) genes (*rnL* and *rnS*), and one tRNA gene (*trnM*). Consistent with previous studies, *P. inutile* shares the same gene arrangement as other *Pleurocorallium* species, while exhibiting distinct gene arrangements compared to closely related genera, such as *Corallium* and *Hemicorallium*. A phylogenetic tree based on protein-coding and rRNA genes from currently available Coralliidae species indicated that *Pleurocorallium* exhibited greater within-genus genetic distances than *Corallium* and *Hemicorallium*.

**Conclusions:**

Our study clearly distinguished *P. inutile* from other species using published mitochondrial genomes. This sequence information will be useful for accurate biodiversity assessments and understanding octocoral mitochondrial gene evolution in the future.

**Supplementary Information:**

The online version contains supplementary material available at 10.1007/s11033-025-10583-3.

## Introduction

**Fig. 1:**
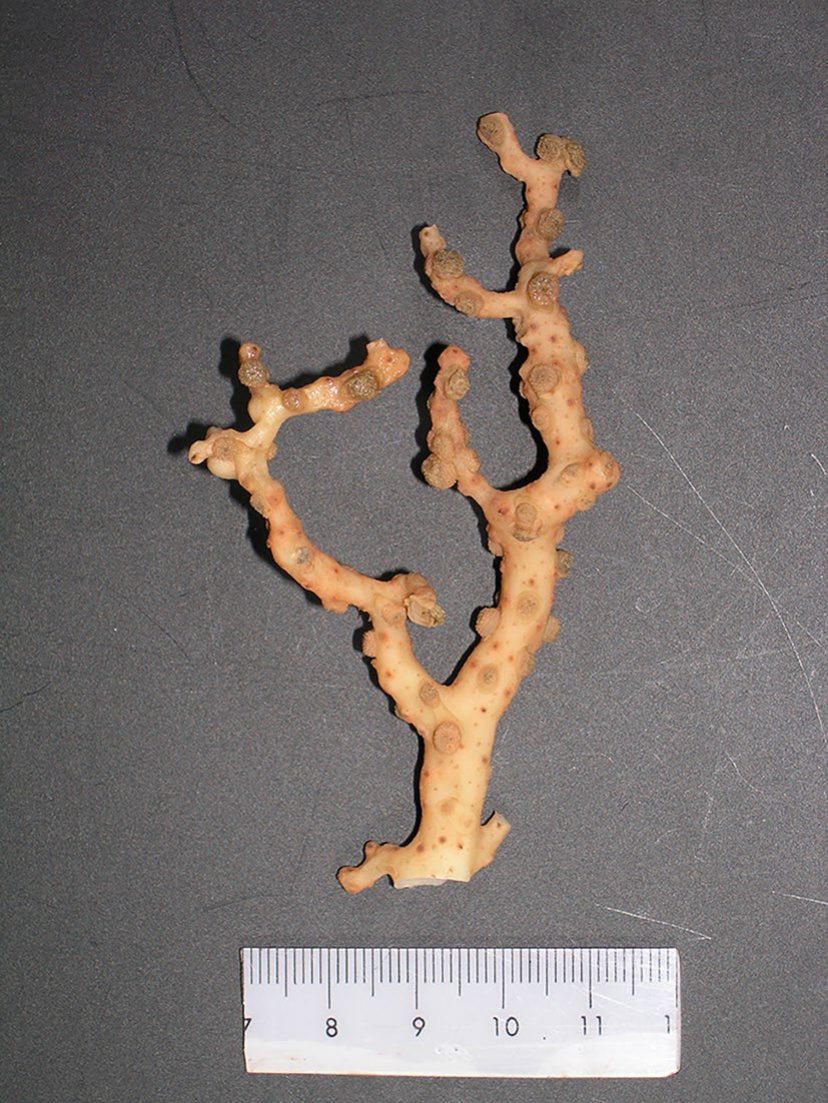
Picture of *Pleurocorallium inutile* (OCA-Cn20071115-016)

Three genera of octocorals within the family Coralliidae (*Corallium, Pleurocorallium*, and *Hemicorallium*) are used as jewelry due to their hard axial skeletons. They are commonly referred to as “precious coral” [1], as they have high commercial value. They are harvested in regions such as the Mediterranean, Japan, Taiwan, and Hawaii [2]. The genus *Pleurocorallium* (17 species) contains economically valuable species in Japan, such as *Pleurocorallium konjoi* (Kishinouye, 1903) and *P. elatius* (Ridley, 1882). Because of the drastic decline in the harvestable quantities of precious corals in recent years, sustainable use and management of these resources have become necessary. Some of these species have been proposed for trade regulation under the Convention on International Trade in Endangered Species of Wild Fauna and Flora [3, 4]. Despite their importance, identifying species status within *Pleurocorallium* remains challenging. Genetic information on this genus is relatively limited and previous studies have demonstrated that eight mitochondrial and elongation factor genes cannot resolve species boundaries between *P. konjoi* and *P. elatius* [1, 5]. Therefore, it is important to accumulate more genetic data to identify *Pleurocorallium* species status and boundaries for the sustainable management of each species. *P. inutile* Kishinouye, 1902 (Fig. 1) has been reported from Kochi, Japan [6] to Taiwan [7], and since it inhabits depths of 245 m [8], its habitat distribution overlaps with that of other precious coral species. In Japan, *P. inutile* is called damesango, meaning “useless coral”, because of its low commercial value as jewelry. The phylogenetic status of *P. inutile* remains ambiguous because eight mitochondrial (*16S*, *ND1*, *ND2*, *ND3*, *ND6*, *COI*, *MSH*, and *IGR1*) and elongation factor genes cannot discriminate between *P. inutile* and *P. thrinax* (Bayer & Stefani, 1996) [1].

Low-coverage genome sequencing (genome skimming) is a promising method for obtaining genetic information, including for reconstructing complete mitochondrial genomes [9]. In this study, to better understand the species status and phylogenetic relationships between the genus *Pleurocorallium* and the evolutionary process of octocoral mitochondrial genomes, we determined the complete mitochondrial genome of *P. inutile* using genome skimming.

## Materials and methods

In November 2007, a specimen of *P. inutile* was collected from a depth of 245 m near Tanegashima Island, Japan. The specimen was purchased from a legal fishing company, and a portion of the specimen was preserved at room temperature in 99% ethanol. The exact sampling locations were not disclosed to prevent illegal harvesting of precious corals. Nonaka and Muzik [8] have provided detailed morphological observations. Sequence reads were obtained using the MiSeq platform with the MiSeq Reagent kit v3 (2 × 300 bp; Illumina, San Diego, CA, USA) after extraction of genomic DNA using the DNeasy Blood & Tissue Kit (Qiagen, Hilden, Germany). Genomic DNA libraries were prepared using the Nextera Prep Kit (Illumina), according to the manufacturer’s instructions. After trimming adapters using Fastp [10], bacterial and human-derived reads were subsequently removed using Kraken2 [11] and the minikraken2_v1 database. The ‘extract_kraken_reads.py’ script was used with –exclude -t 9606, 2’ options. When analyzing genomes with relatively small sizes and a largely conserved gene arrangement, such as mitochondrial genomes, comparisons with closely related reference genomes can improve analytical efficiency and are useful for handling true repeat sequences, as well as detecting large deletions and insertions [12]. Therefore, we performed a reference-based assembly. The remaining reads were assembled using NOVOPlasty [13] using the complete mitochondrial genome data of *P. elatius* (accession number: AB700134) as the reference genome. Gene annotation was performed using MITOS2 [14]. The maximum likelihood phylogenetic tree among the three genera, *Pleurocorallium*, *Hemicorallium*, and *Corallium*, was inferred using IQTREE version 2.1.4 [15]*.* Seventeen nominal species of *Pleurocorallium*, 22 nominal species of *Hemicorallium*, and 7 nominal species of *Corallium* have previously been reported. We used five species of *Pleurocorallium* (including *P. inutile*), three species of *Hemicorallium*, and two species of *Corallium* for which completely annotated mitochondrial genomes are available. *Briareum asbestinum* (Pallas, 1766) (KC008073) was selected as the outgroup, and each of the 14 protein-coding and ribosomal RNA (rRNA) genes was individually aligned based on the *P. inutile* sequences. Following the method described by Uda et al. [2], the gene order and orientation of *Corallium*, *Hemicorallium*, and *Pleurocorallium* were adjusted to match those of *Briareum*, and a concatenated alignment totaling 18,379 bp was used for phylogenetic analysis (Fig. S1 and Table S1). The alignment was partitioned based on gene regions, with protein-coding genes further divided by codon position (1st, 2nd, and 3rd codon sites), and rRNA genes were treated as separate partitions. The best-fit evolutionary models for each partition were determined using ModelFinder Plus (MFP + MERGE) [16], which automatically selects the most appropriate substitution model for each partition and merges similar partitions to optimize model fitting. Bootstrap analysis was performed with 1000 replicates using the UFBoot Ultrafast Bootstrap Approximation [17]. Single-branch tests were also performed with 1000 replicates using SH-aLRT, a Shimodaira–Hasegawa-like approximate likelihood ratio test [18]. The final tree was constructed using FigTree version 1.4.4 [19].

## Results and discussion

**Fig. 2:**
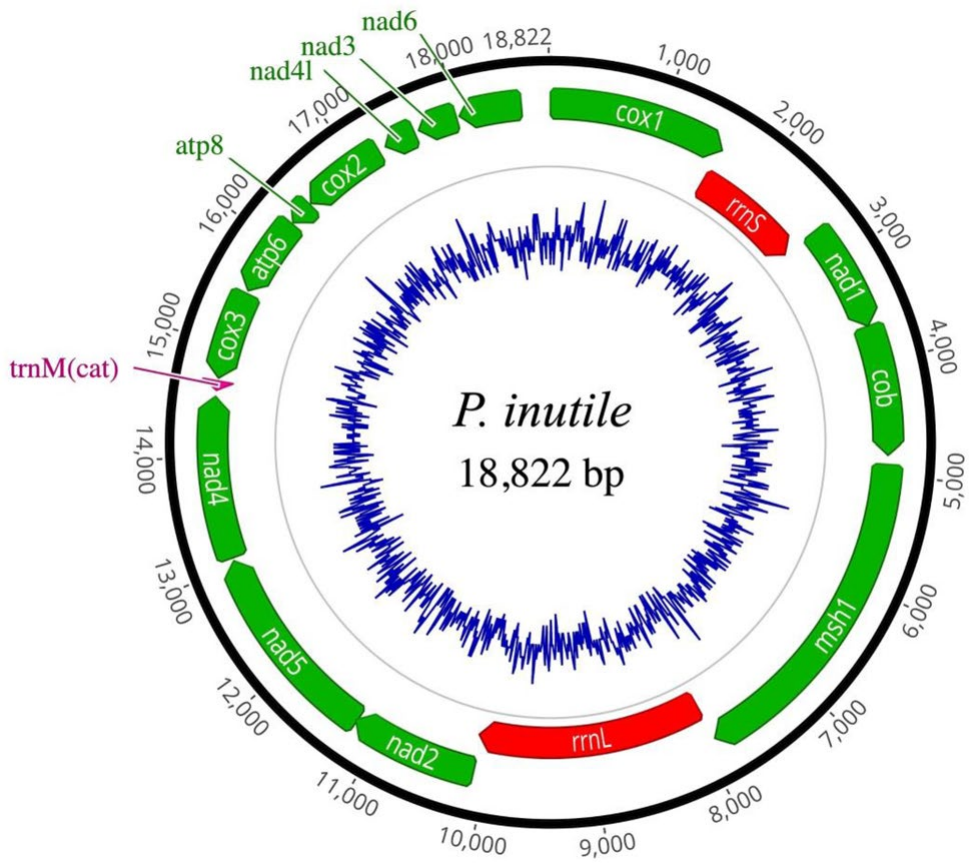
Map of the complete mitochondrial genome of *P. inutile* in this study drawn using Geneious Prime 2024.0.7 (https://www.geneious.com). The blue line shows the GC content

A total of 3,572,760 raw reads were obtained for *P. inutile*. The raw sequencing reads were deposited in the DNA Databank of Japan under Accession Number DRA020369. A total of 3,332,438 reads remained after filtering using Fastp and Kraken2. The percentage of reads derived from the mitochondria was 0.1%, and the average read coverage was 51 (Fig. S2). The total length of the complete mitogenome of *P. inutile* was 18,822 bp and the base composition of each nucleotide was 29.9%, 18.2%, 20.0%, and 31.9% for A, C, G, and T, respectively. Consistent with other mitochondrial genomes of Coralliidae, there were 14 protein-coding genes, two rRNA genes (*rnL* and *rnS*), and one tRNA gene (*trnM*) (Fig. 2). *P. inutile* was found to be closely related to *P. elatius* and *P. konojoi* within the genus *Pleurocorallium*, but it formed a distinct clade, separate from these and other species in the genus. The gene arrangement of *P. inutile* was consistent with that of other *Pleurocorallium* species (at least with that of *P. konojoi, P. elatius, P. secundum* [Dana, 1846]*,* and *P. porcellanum* [Pasernak, 1981]). However, unlike gene arrangements in the genera *Corallium* and *Hemicorallium*, a unique gene arrangement was observed in which the arrangements of *msh*, *rrnL*, *nad2*, *nad5*, *nad4*, *trnM*, *cox3*, *atp6*, *atp8*, and *cox2* were inverted, as reported by Uda et al. [2].

**Fig. 3:**
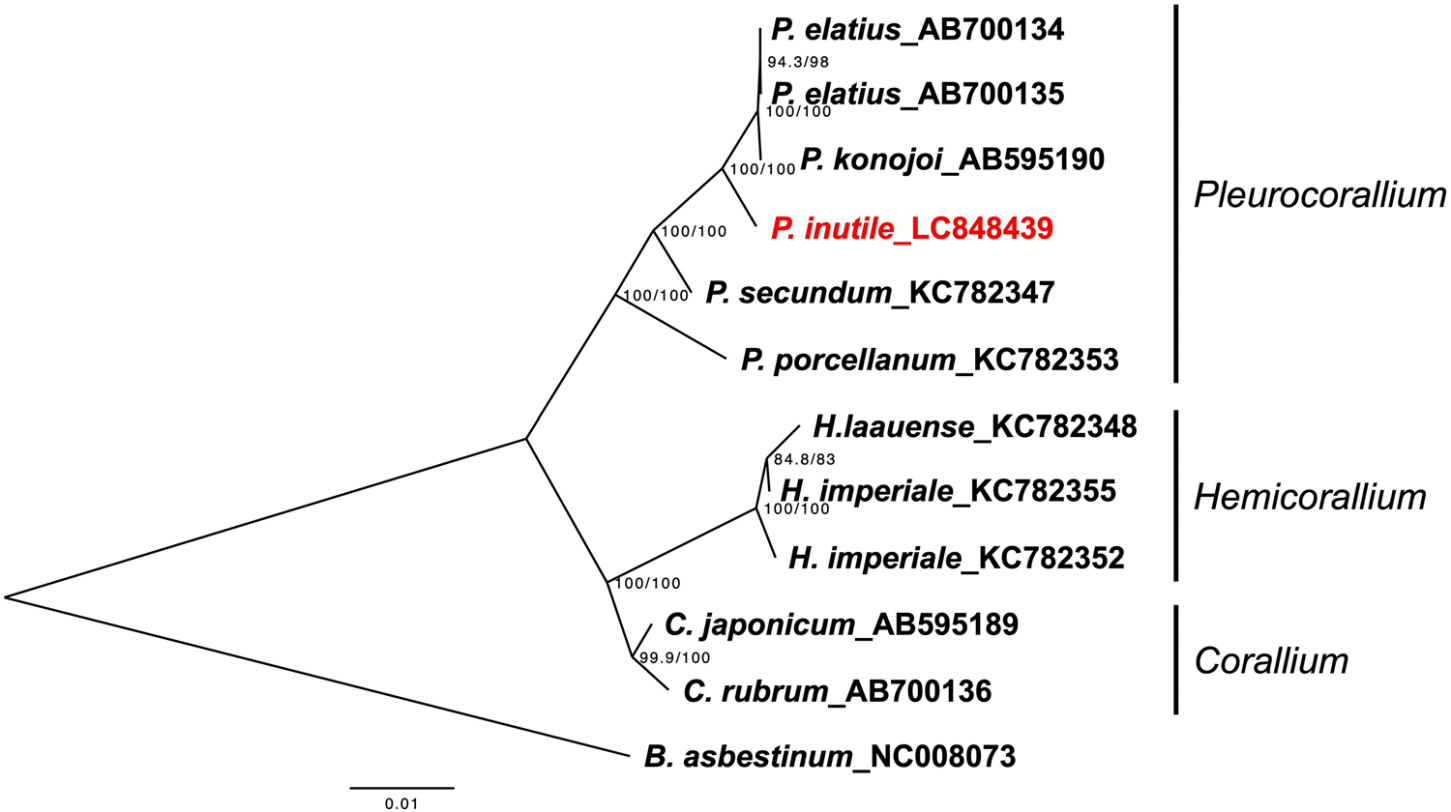
Phylogenetic three of Coralliidae genera for which annotated complete mitogenomes are currently available. The following sequences were used: AB700134, AB700135, AB700136 [20], AB595189, AB595190 [2], KC782353, KC782347, KC782348, KC782352, KC782355 [21], NC008073 [22]. Branch lengths are relative to genetic divergence, and values on each node represent SH-aLRT/ultrafast bootstrap values. Node supports are provided for SH-aLRT values ≧ 80% or ultrafast bootstrap values ≧ 95%

The phylogenetic tree also revealed that the genus *Pleurocorallium* exhibited greater within-genus genetic distances than the other two genera, and the genetic distance between *P. elatius* and *P. porcellanum* (Fig. 3) was as distinct as that between *Corallium* and *Pleurocorallium* (Fig. 3).

Mitochondrial gene arrangement between the genus *Pleurocorallium* and other genera has also been reported [2, 21]. Tu et al. [1] noted that *P. inutile* is genetically closest to *P. thrinax* and that eight mitochondrial genes and elongation factor genes do not distinguish between them, pointing to the importance of using more genetic regions. *P. thrinax* is known to occur in the vicinity of Papua New Guinea [23], and although we were unable to obtain a sample, it may be possible to identify the species in the future by determining the complete mitochondrial gene region of *P. thrinax* and comparing it with the sequences obtained in this study.

*P. inutile* was first described as a new species in Kochi, Japan, in 1902 [6], but coral fisheries had already been operating prior to that, and it is likely that this species was caught as a bycatch even earlier. Although its distribution has been confirmed in Taiwan [7], the only verified occurrence within Japan was reported by Nonaka and Muzik [8]. Although this species is expected to inhabit areas ranging from Japan to Taiwan, a low population density may explain the scarcity of additional records. Because of the lack of confirmed records, how much of this “useless coral” is caught as bycatch with valuable coral during harvesting and the total amount of the resource are unclear. It is desirable to accurately assess harvest volumes for each species using genetic markers and to conduct monitoring using environmental DNA, which has recently shed light on the dynamics of deep-sea environments [24].

## Conclusion

In this study, we report the complete mitochondrial genome sequence of *P. inutile*. Among the reported mitochondrial genomes, it is most closely related to *P. konojoi* and *P. elatius*. The gene arrangement of *P. inutile* was consistent with that of other *Pleurocorallium* species, but differed from the arrangement observed in the genera *Corallium* and *Hemicorallium*. The mitochondrial genome reported in this study provides foundational data for future efforts, enabling accurate species identification, and contributing to the sustainable management of coral resources.

## Supplementary Information

Below is the link to the electronic supplementary material.

Supplementary file1 (DOCX 118 KB)

Supplementary file2 (DOCX 79 KB)

## Data Availability

The mitochondrial genome sequence data supporting this study’s findings are openly available in DDBJ under the Accession No. LC848439. In addition, the raw sequencing data are deposited in DDBJ under the Accession No. DRA020369.
