# Supplementary material for: The complete mitochondrial genome of Pleurocorallium inutile (Octocorallia: Scleralcyonacea: Coralliidae)
Source: Mol Biol Rep. 2025 Jun 1;52(1):531. doi: 10.1007/s11033-025-10583-3 (PMC12127222; doi:10.1007/s11033-025-10583-3)
Supplement: Supplementary file 1 — Supplementary file1 (DOCX 118 KB) [file 11033_2025_10583_MOESM1_ESM.docx]

**Supplementary Information**

**Journal:** *Molecular Biology Reports*

**The complete mitochondrial genome of *Pleurocorallium inutile* (Octocorallia: Scleralcyonacea: Coralliidae)**

Kenji Takata^1^, Masanori Nonaka^2^, Fei Xia^3^, Taisei Kikuchi^3^, Kodai Gibu^1^, Nina Yasuda^1^

1, Graduate School of Agricultural and Life Sciences, The University of Tokyo, Yayoi, Tokyo, Japan

2, Okinawa Churashima Foundation Research Institute, Motobu, Okinawa, Japan

3, Graduate School of Frontier Sciences, The University of Tokyo, Kashiwa, Chiba, Japan

**Corresponding author**

Nina Yasuda: [ninayausda@gmail.com](mailto:ninayausda@gmail.com)

Fig. S1| Nucleotide length of each gene and the process of aligning and concatenating genes in the order and orientation matching that of *Briareum*.


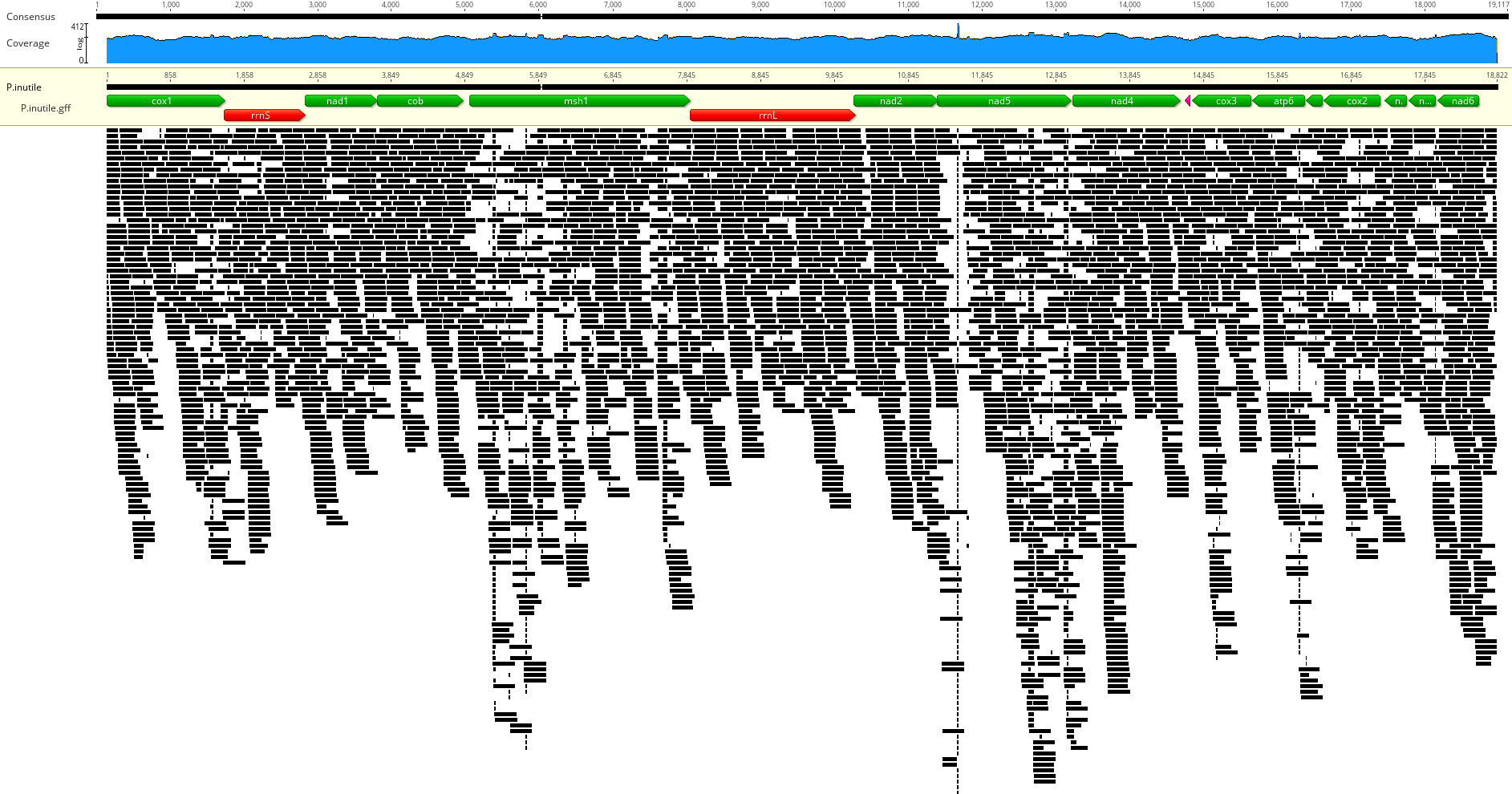


Fig. S2| Read coverage depth map of *Pleurocorallium inutile* created using Geneious Prime 2024.0.7 (<https://www.geneious.com>).
